# Takotsubo Cardiomyopathy Mimicking Stent Thrombosis After Percutaneous Coronary Intervention

**DOI:** 10.1177/2324709618773793

**Published:** 2018-05-02

**Authors:** Furqan Khattak, Muhammad Khalid, Ghulam Murtaza, Timir K. Paul

**Affiliations:** 1 East Tennessee State University, Quillen College of Medicine, Johnson City, TN, USA

**Keywords:** takotsubo cardiomyopathy, myocardial infarction, apical ballooning, left ventricle apex akinesia

## Abstract

Takotsubo cardiomyopathy, also known as “broken heart syndrome,” is a transient left ventricular dysfunction associated with stress (usually emotional) induced myocardial injury and stunning. It often presents as myocardial infarction on surface electrocardiogram (EKG). Diagnosis is made by coronary angiography, which rules out coronary artery disease and shows pathognomonic apical ballooning. In this article, we present a case of a 72-year-old woman who initially presented with an ST segment elevation myocardial infarction on EKG. Coronary angiography showed severe left anterior descending artery and diagonal lesions requiring percutaneous coronary intervention. Post–percutaneous coronary intervention, EKG changes resolved. The next day, the patient developed recurrent chest pain and her EKG showed diffuse T-wave inversion in precordial leads with reemerging ST segment elevations concerning for stent thrombosis. The patient underwent repeat emergent coronary angiography, which showed patent stents and findings consistent with takotsubo cardiomyopathy.

## Introduction

Takotsubo cardiomyopathy (TC), also known as “stress cardiomyopathy” or “broken heart syndrome,” is a transient syndrome of left ventricular apical akinesis, which often mimics acute ST segment elevation myocardial infarction (STEMI). Patients usually present after an emotionally stressful event with chest pain and findings of elevated cardiac enzymes and/or ST segment elevation on surface electrocardiogram (EKG). However, coronary angiography excludes significant coronary artery stenosis and shows left ventricular (LV) apical ballooning. In this article, we present an interesting case of TC post–percutaneous coronary intervention (PCI) masquerading as stent thrombosis.

## Case Description

**Figure 1.**
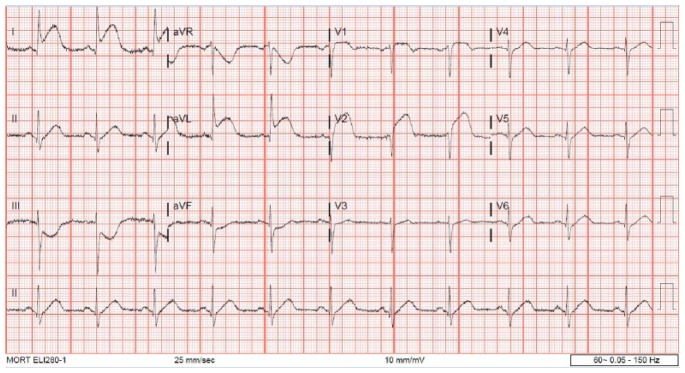
Initial EKG showing ST elevation in leads 1, aVL, V1, V2, and ST depressions in inferior leads.

**Figure 2.**
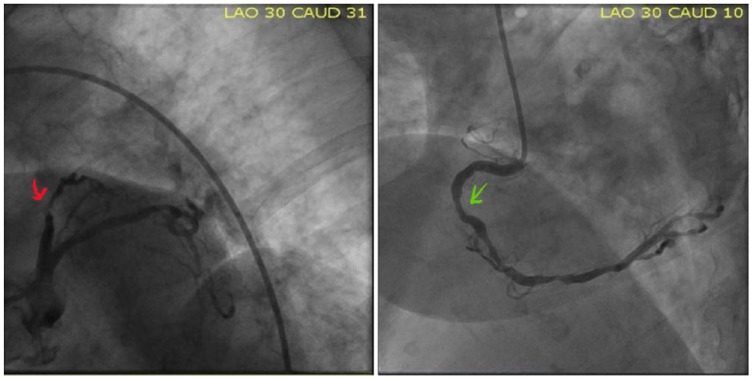
Coronary angiography on presentation showed severe disease in LAD (red), D1 and nonobstructive diffuse disease in RCA (green).

**Figure 3.**
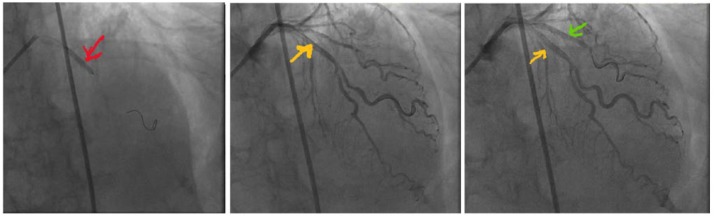
(a) Stent deployment in LAD (red), (b) Stent in LAD (yellow arrow), and (c) Stents demonstrated in LAD and D1 (green).

**Figure 4.**
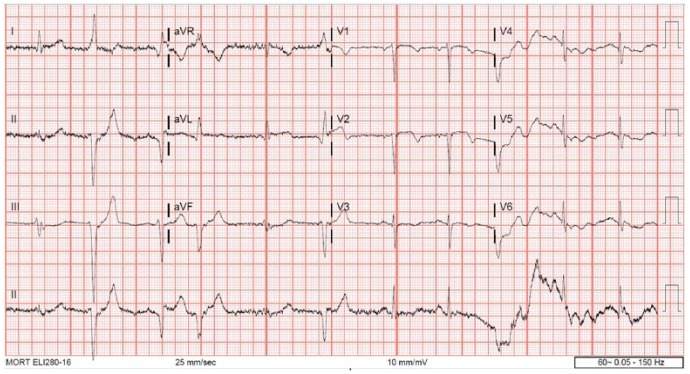
Post-PCI resolution of ischemic changes on EKG.

**Figure 5.**
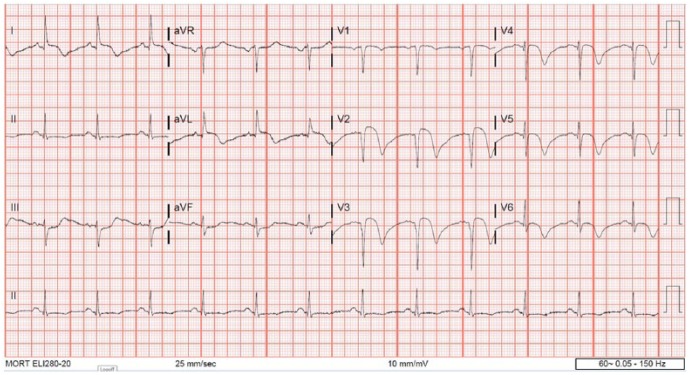
EKG on day 2 showing ST elevations in leads 1, V2, V3 with ST depressions in inferior leads and diffuse TWI in precordial leads.

**Figure 6.**
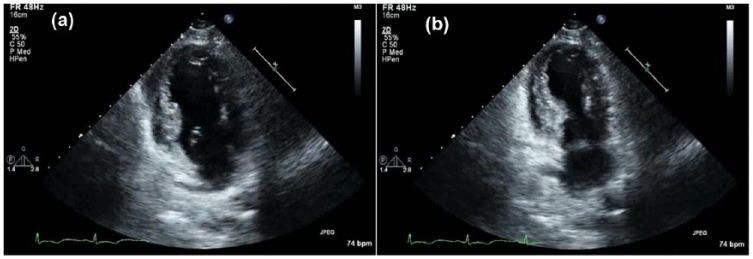
Echocardiogram demonstrating apical hypokinesis: (a) ventricular diastole and (b) ventricular systole.

**Figure 7.**
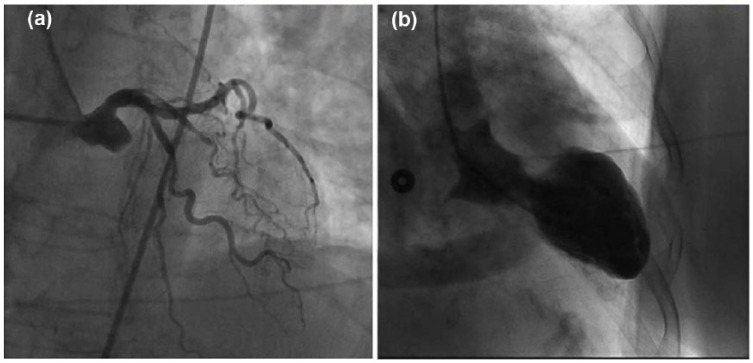
(a) Repeat coronary angiography demonstrating patent stents and (b) Ventriculography showing apical ballooning.

A 72-year-old woman with history of hypertension, hyperlipidemia, and partial nephrectomy presented to hospital with sudden onset of retrosternal chest pain radiating across the precordium, while narrating the story of her grandkid’s ordeal in prison to a friend. Pain was associated with shortness of breath, nausea, and profuse sweating. On presentation, she was afebrile and hemodynamically stable with a blood pressure of 148/78 mm Hg, pulse of 63 beats per minute, and respiratory rate of 18 breaths per minute. Physical examination was normal save for anxiousness and nervousness. Laboratory workup showed normal complete blood count, basic metabolic panel, and mildly elevated troponin level at 0.06. EKG revealed ST segment elevation in leads I, aVL and V2 with reciprocal changes in inferior leads concerning for anterolateral infarction (Figure 1). The patient was emergently taken to the catheterization laboratory where coronary angiography demonstrated 95% lesions in left anterior descending artery (LAD) and first diagonal artery (D1) requiring PCI with drug-eluting stents (Figures 2 and 3). Post-PCI, EKG showed resolution of ST changes (Figure 4), and echocardiogram showed normal LV ejection fraction (EF). On day 2, the patient developed recurrent chest pain. Repeat EKG showed diffuse T-wave inversions in precordial leads, reemerging ST elevations in 1, V2, V3 with reciprocal ST segment depressions in inferior leads and troponin elevation up to 12 concerning for re-infarction (Figure 5). A stat bedside transthoracic echocardiogram revealed ballooning/akinesia of the LV apex and mildly reduced EF of 45% to 50% (Figure 6a and b). The patient was taken for repeat emergent coronary angiogram to rule out stent thrombosis. Coronary angiogram showed widely patent stents in LAD and D1. Left ventriculogram showed apical ballooning consistent with TC (Figure 7). It remains unclear if her initial presentation was from TC with incidental discovery of coronary artery disease or if she developed TC after PCI.

## Discussion

TC is a transient reversible LV dysfunction usually associated with emotional stress. It was first reported in a Japanese patient in 1983 by Sato et al.^1^ The incidence of TC is about 1% to 2% among patients presenting with myocardial infarction. It has been reported more frequently in postmenopausal women aged >55 years.^2,3^ In 2006, the American Heart Association classified TC as an acquired cardiomyopathy.^4^ Diagnosis is based on Mayo criteria^5^ and imaging coronary angiography to rule out coronary artery disease. Mayo clinic diagnostic criteria include the following: (1) transient hypokinesia, akinesia, or dyskinesia of the mid segment of the left ventricle with or without apex involvement; (2) no obstructive atherosclerotic coronary artery disease or evidence of plaque rupture on coronary angiogram; (3) new EKG findings (ST elevation/T wave inversion) or mild troponins elevation; and (4) no pheochromocytoma or myocarditis. All 4 criteria need to be met to make a diagnosis of TC. Exclusion of pheochromocytoma is not routinely performed, which would require measurement of serum and urine metanephrines.

Patients with TC commonly present with chest pain and shortness of breath and usually recall a preceding emotional or physical stress as a trigger. Hemodynamic instability with hypotension is rare. EKG may show ischemic changes and laboratory workup may show elevated cardiac enzymes. The most common EKG abnormality is ST segment elevation mimicking myocardial infarction.^6^ Kosuge et al^7^ compared EKG findings of TC with STEMI in patients admitted within 6 hours of symptom onset and reported that a combination of ST segment depression in lead aVR and absence of ST segment elevation in lead V1 had a 91% sensitivity, 96% specificity, and 95% accuracy to diagnose TC. Mugnai et al^8^ reported that the absence of abnormal Q waves, presence of ST segment depression in aVR, and the lack of ST segment elevation in V1 were significantly associated with TC with a combined specificity of 95% and positive predictive value of 85.7%. Tamura et al^9^ reported that TC had a much lower prevalence of ST segment elevation of ≥1 mm in lead V1 compared with anterior STEMI measured at the J point. Jim et al^10^ reported that absence of reciprocal changes in inferior leads in patients presenting with anterior wall myocardial infarction favors a diagnosis of TC. Our patient presented on the day after a STEMI with diffuse T wave inversion across the precordium. Her EKG did not meet any specific criteria for TC mentioned above, but coronary angiogram showed classic apical ballooning with clean coronary arteries consistent with TC, distinct from the event she had experienced the previous day. Alternatively, her presentation may reflect post-infarction pericarditis, or an intracranial event. Our patient had neither a friction rub, nor evidence for an intracranial event ruling out these alternative differentials.

TC has different variants on echocardiogram, namely, classical, inverted, or midventricular patterns. The characteristic features of classic TC on echocardiogram include apical hypokinesia/ballooning and basal hyperkinesia. Inverted TC presents with ST segment elevation in inferior leads, normal EF, apical hyperkinesia, and basal to midventricular ballooning on echocardiogram. Midventricular pattern characteristically shows akinesia of the midventricular segment with or without involvement of basal and apical segment on echocardiogram.^11,12^

Various mechanisms have been postulated to explain TC including acute coronary syndrome with spontaneous reperfusion, coronary microvascular dysfunction, and catecholamine-mediated myocardial dysfunction.^13^ The association of subarachnoid hemorrhage and TC called “neurological stunning” consists of features of brain injury, TC, and absence of coronary artery disease. Effect of catecholamines on the adrenergic innervation of the heart may explain the transient myocardial dysfunction in TC. Animal models have suggested that activation of adrenergic receptors is the main trigger for stress induced myocardial injury leading to regional LV wall motion abnormalities.^14^

Initial management of TC is like myocardial infarction until definitive diagnosis is made. Treatment is supportive with diuretics, angiotensin converting enzyme inhibitor, and β-blockers. β-Blockers are used to control the excessive effect of catecholamine but should be started once pulmonary edema, if present, is resolved, as per heart failure guidelines. Other heart failure agents can be considered as clinically indicated. Congestive heart failure is the most common complication reported and occurs in 20% of patients with TC,^2^ especially with right ventricular involvement.^15^ The transient systolic dysfunction associated with TC is usually reversible within 4 to 8 weeks and alternate diagnosis should be considered if cardiomyopathy does not resolve on repeat echocardiogram. A study by Chao et al^16^ showed that a significant number of patients with LAD occlusion on coronary angiography and STEMI have associated TC features.

## Conclusion

This case highlights an interesting association between coronary artery disease and TC leading to repeat angiography for concerns of stent thrombosis. TC should be considered as one of the main differential diagnosis of ST segment elevation, especially in postmenopausal women with obvious emotional or physical stress. TC post-STEMI is an uncommon presentation but should be considered as a differential along with post-infarction pericarditis, and an intracranial event. Patients must be followed once TC is diagnosed, as the condition may be reversible in 4 to 8 weeks when placed on appropriate medications.
